# Vitamin D supplementation at different doses affects the vagal component of the baroreceptor reflex and the Bezold-Jarisch reflex in eutrophic rats

**DOI:** 10.3389/fphys.2022.934625

**Published:** 2022-08-05

**Authors:** Alexandre C. Fioretti, Nuha A. Dsouki, Barbara do Vale, Rodrigo P. de Carvalho, Daniel P. M. Dias, Daniel P. Venancio, Fernando L. A. Fonseca, Monica A. Sato

**Affiliations:** ^1^ Department of Morphology and Physiology, Centro Universitario FMABC, Santo Andre, Brazil; ^2^ Centro Universitario Barao de Maua, Ribeirao Preto, Brazil; ^3^ Laboratory of Clinical Analysis, Department of Pathology, Centro Universitario FMABC, Santo Andre, Brazil

**Keywords:** vitamin D, arterial pressure, heart rate, cardiovascular, autonomic regulation, heart rate variability

## Abstract

Vitamin D has been used to prevent several diseases. The 1,25 (OH) 2D3, the active form of vitamin D (VitD), participates in calcium metabolism, and has direct action in various tissues as those of the cardiovascular system binding to the VitD receptor. We investigated whether the supplementation with different doses of VitD affect or not the resting mean arterial pressure (MAP) and heart rate (HR), heart rate variability (HRV), baroreceptor and Bezold-Jarisch reflexes in eutrophic rats. Adult male Wistar rats were randomly assigned in 4 groups (Control, VitD 15, 250, and 3,750 IU/day, *n* = 6/group). After 3 days of supplementation, MAP and HR recordings were performed in freely moving rats. Baseline (resting) MAP, HR, and HRV showed no difference in Control and VitD groups. Nevertheless, the index of the baroreceptor reflex showed that the bradycardic component of the baroreflex evoked by a pressor dose of phenylephrine (3 μg/kg of b.w.) in bolus injection had a significant increase in rats supplemented with VitD 15 IU/day for 3 days compared to Control animals. No difference was observed in the index of the baroreflex evaluated with phenylephrine in rats treated with VitD 250 and 3,750 IU/day for 3 days in comparison to the Control group. The index of the baroreceptor reflex evaluated with an intravenous bolus injection of a depressor dose of sodium nitroprusside (30 μg/kg of b.w.) showed that the tachycardic component of the baroreflex is not different comparing all groups supplemented with VitD and Control animals. Rats supplemented with VitD 15 IU/day presented exaggerated bradycardic responses to the intravenous injection of phenylbiguanide (PBG, 5 μg/kg of b.w.) compared to Control animals, despite the similar hypotension in both groups. Higher doses of supplementation of VitD (250 and 3,750 IU/day for 3 days) abolished the hypotension and bradycardia induced by PBG. The findings suggest that the supplementation with different doses of VitD (15, 250, and 3,750 IU/day) for 3 days did not affect the resting arterial pressure, heart rate and autonomic modulation on the heart in rats. Despite that, the supplementation with a low dose of VitD (15 IU/day for 3 days) improved the sensitivity of the bradycardic component of the baroreflex, whereas higher doses of supplementation with VitD (250 and 3,750 IU/day for 3 days) were unable to cause such effect. In addition, the Bezold-Jarisch reflex responses can be affected regardless the dose of VitD (15, 250 or 3,750 IU/day) supplementation for 3 days in rats.

## 1 Introduction

Vitamin D (VitD) has gained a growing focus in the clinics in different countries over the last years. Despite this steroid prohormone is synthesized in the skin following ultraviolet exposure, it can also be obtained by supplemental or dietary intake (Ke et al., 2015; Pilz et al., 2016). In circulation, VitD sterols are mainly transported by the vitamin D-binding protein (DBP), which is a serum glycoprotein secreted by the liver. Vitamin D itself is biologically inactive, nevertheless when pass in the liver is hydroxylated to 25-hydroxyvitamin D [25(OH)D], which in turn, is hydroxylated in the kidney resulting in the formation of the active VitD hormone known as 1,25(OH)_2_D or calcitriol (Christakos et al., 2016; Pilz et al., 2016). The actions of VitD are dependent on the binding to Vitamin D Receptors (VDR) in the cytoplasm of cells expressed throughout the body in different tissues such as the skeletal, cardiac or smooth muscle (Wang and DeLuca, 2011; Christakos et al., 2016; Pilz et al., 2016), kidney, and activated T cells (Bhalla et al., 1983; Christakos et al., 2016).

Several studies have shown that low levels of serum calcitriol increase the risk of various morbidities (Zittermann et al., 2012; Dror et al., 2013). Vitamin D deficiency has been correlated with cancer, cardiovascular disease, autoimmunity, diabetes mellitus, osteoarthritis, depression, and lung dysfunction (Garland et al., 2014; Christakos et al., 2016; Mirhosseini et al., 2016; Pilz et al., 2016) Although previous reports have investigated the consequences of VitD deficiency in the human body, the effects of hypervitaminosis D are still not fully understood. On the other hand, the toxicity of VitD in humans is rare and usually caused by taking high doses of vitamin D due to misuse of supplements or erroneous prescriptions (Asif and Farooq, 2021).

Currently, the ease to purchase the VitD by the general population and the absence of regulatory process to control the VitD prescription have provided the conditions for an excessive supplementation with VitD, which could implicate in a growing risk for health and result in toxicity (Sharma et al., 2017; Rahesh et al., 2020). The most often described clinical symptoms of vitamin D toxicity include confusion, apathy, recurrent vomiting, abdominal pain, polyuria, polydipsia, and dehydration, which are symptoms related to severe hypercalcemia (Vieth, 1990; Marcinowska-Suchowierska et al., 2018; Rahesh et al., 2020). Three hypotheses have been raised to explain the VitD toxicity: 1) high concentration of VitD metabolites exceed the Vitamin D Binding Protein (DBP) transport capacity and cause a release of free 1,25(OH)_2_D (Marcinowska-Suchowierska et al., 2018); 2) increased serum concentration of the active VitD form leads to an increased intracellular concentration (Selby et al., 1995; Marcinowska-Suchowierska et al., 2018); 3) in hypervitaminosis D, the concentration of various VitD metabolites, particularly 25(OH)D, enter the cell nucleus, consequently its high concentration with strong affinity for VDR stimulates the transcription process in the cells (Bikle et al., 1986; Marcinowska-Suchowierska et al., 2018).

One of the main concerns related to the high circulating levels of 1,25-dihydroxyvitamin D is associated with the increased risk of hypertension (van Ballegooijen et al., 2015). Nevertheless, it is still unknown if this risk is only dependent on direct actions of VitD in the arterial wall causing vasoconstriction and increase in the blood pressure or any change could be yielded in the autonomic nervous system control leading to an imbalance in the cardiovascular regulation. Thereby, in this study, we focused to investigate whether the supplementation with different doses of VitD affect or not the resting cardiovascular variables (arterial pressure and heart rate) and the cardiac autonomic modulation in conscious rats. We also evaluated the responsiveness of the baroreceptor and cardiopulmonary (Bezold-Jarisch) reflexes in order to verify the effects of supplementation of VitD upon stimulation conditions of the cardiovascular system. Further, the immunolabeling of VDR in the right atrium was accomplished to understand if the cardiac cells were affected by the treatment with different doses of VitD.

## 2 Materials and methods

### 2.1 Animals

Adult male Wistar rats (300–350 g, *N* = 6/group) obtained from the central animal facility of the Faculdade de Medicina do ABC, Centro Universitário FMABC (CUFMABC) were used. Rats were housed in groups of 3 in plastic cages in an air-conditioned room (20°C–24°C) with a 12:12-h light-dark cycle and had free access to standard chow pellets and water. All procedures were performed in accordance with the National Institutes of Health (NIH) Guide for the Care and Use of Laboratory Animals and were approved by the Animal Ethics Committee of the FMABC/CUFMABC (protocol number # 10/2018).

### 2.2 Vitamin D supplementation

Eutrophic rats were randomly divided in 4 groups with 6 rats each and submitted to oral supplementation with Vitamin D (VitD) or vehicle (condensed milk) during 3 days:

Control Group: Only vehicle (condensed milk, *n* = 6)
VitD15 Group: Vit D 0,015 mg/day (equivalent to 15 UI/day, *n* = 6)
VitD250 Group: Vit D 0,25 mg/day (equivalent to 250 UI/day, *n* = 6)
VitD3750 Group: Vit D 3,75 mg/day (equivalent to 3750 UI/day, *n* = 6)

Doses of VitD ranged from deficiency to toxic levels as previous reported by the study of Mirhosseini et al. (2016).

### 2.3 Surgical Preparation and arterial pressure and heart rate recordings in free moving rats

Twenty-four hours before the experiment, rats were anesthetized with 2% isoflurane in 100% O_2_ for the cannulation of the femoral artery for pulsatile arterial pressure (PAP), mean arterial pressure (MAP) and heart rate (HR) recordings in a data acquisition system (Power Lab 16SP, AD Instruments, New South Wales, AU). The HR signals were derived from PAP. The femoral vein was also cannulated for infusion of drugs.

The catheters used for cannulation of the blood vessels consisted of a polyethylene tubing (PE-50 with 15 cm length connected to a PE-10 with 2.5 cm length, Clay Adams, NJ, United States). The tip of the PE-10 tubing was placed into the femoral artery and vein through a small incision in the distal portion of the blood vessels and inserted until almost reach out the aortic artery and inferior vena cava, respectively. Catheters were filled with heparinized saline (50–100 IU/ml), the outlet of the cannula was obstructed to avoid leaking and tunneled subcutaneously to exit through a small incision at the neck between the scapulae. Catheters were held with sutures to the surrounding skin and all incisions were suture closed. After surgery, animals were maintained in individual plastic cages with food and water *ad libitum* and allowed 24 h to recover before the experiments.

On the day of the experiment, the animals were placed in a quiet experimentation room of the lab and allowed a minimum of 60 min of habituation before starting the recording in free moving condition. The arterial catheters were connected to a PE-50 tubing connected to a transducer (AD Instruments, New South Wales, AU). The pulsatile arterial pressure (PAP), mean arterial pressure (MAP), and heart rate (HR) signals were amplified and digitalized in a Power Lab 16SP System (AD Instruments, New South Wales, Australia).

### 2.4 Heart rate variability analysis

Baseline arterial pressure recordings for at least 30 min were processed by computer software that applies an algorithm to detect beat-to-beat inflection points of a periodic waveform, determining beat-by-beat values of systolic and diastolic arterial pressures. Beat-by-beat pulse interval (PI) series were also generated by measuring the length of time between adjacent diastolic pressure readings, and they were used to determine HRV using the CardioSeries^®^ software. The overall variability of PI was assessed by the variance of the time series. All-time series were obtained from each experimental group (control and VitD supplementation groups). The PI variabilities in the frequency domain were assessed by spectral analysis as follows: the values of SAP and PI were resampled at 10 Hz by cubic spline interpolation to adjust the time interval between beats. The PI of each segment was examined visually, and segments with artifacts or large transients were excluded.

Each segment of PI was submitted to spectral analysis *via* fast Fourier transform after the Hanning window. The spectra of SAP and PI were integrated into bands of low (LF, 0.2 to 0.75 Hz) and high frequency (HF, 0.75 to 3 Hz) (dos Santos et al., 2010).

For analysis of the PI variabilities in the time domain and the nolinear analysis we used the Kubius software. In time domain time, each segment of PI was submitted to analysis of SDNN and RMSSD, and the case of nonlinear analysis we obtained through geometric analysis: sample entropy, SD1, SD2, α1, and α2.

### 2.5 Evaluation of the baroreceptor and cardiopulmonary (Bezold-Jarisch) reflex in rats submitted to vitamin D supplementation

In order to evaluate the cardiovascular reflexes in free moving rats, PAP, MAP and HR were continuously recorded (baseline) for 30 min. These values were used as a reference to calculate the changes produced by the drug infusions. Baroreceptor reflex responses were evaluated using a pressor dose of i.v. Phenylephrine (PHE, 3 µg/kg, Tocris, Missouri, United States) and a depressor dose of i.v. Sodium nitroprusside (SNP, 30 µg/kg, Tocris, Missouri, United States). The index of baroreceptor reflex was calculated as the maximum ΔHR/maximum ΔMAP at the peak response evoked by PHE and SNP administered in bolus injection. Cardiopulmonary (Bezold-Jarisch) reflex was evaluated using i.v. Phenylbiguanide (PBG, 5 µg/kg, Tocris, Missouri United States) and the cardiovascular responses were evaluated during the next 15 min. All drugs were administrated in a bolus injection.

### 2.6 Plasma calcium and calcitriol determination

A different group of rats from the cardiovascular recordings was used in order to evaluate the plasma calcium and calcitriol. Animals were supplemented with vitamin D in different doses for three consecutive days as described above in item 2.2, and in the fourth day, the rats were deeply anesthetized with 4% isoflurane (Isoforine^®^, Cristalia, Itapira, SP, Brazil) in 100% O_2_ and blood was withdrawn by aorta puncture using a heparinized (5,000 IU/ml) syringe. The blood was transferred to microtubes (Eppendorff^®^) and the samples were centrifuged (2,500 rpm/10 min) under refrigeration (Eppendorff centrifuge^®^) and the plasma was immediately extracted and stored at −80°C for posterior calcium dosage by colorimetric method (Arsenazo III) according to the manufacturer protocol and calcitriol dosage by electrochemiluminescence method. The device used for the analysis was Cobas^®^ 6,000 analyzer series, fully automated (Roche Labs).

### 2.7 Immunohistochemistry labeling of vitamin D receptors in the right atria

The same animals used in the protocol 2.6. were perfused with 40 ml of 10% phosphate buffered formalin solution (Dinâmica^®^) after withdrawal of the blood samples performed in the that protocol. The rats were thoracotomized and the heart was harvest and the right atrium of each heart was maintained in the 10% phosphate buffered formalin solution for 24 h at room temperature. They were then washed several times with 70% (V/V) ethanol, dehydrated in graded ethanol (70%, 80%, 90%, 95%, and 100%) for 1 h in each solution. Afterwards, they were diaphanized twice using 100% xylol solution for 30 min each and finally embedded in liquid paraffin (EP-21-20069, EasyPath^®^). Sections (5 µm) of the right atrium were cut in a microtome (Lupetec MP 2015), mounted in silanized glass slides, maintained overnight at 60^o^C in the incubator, and processed for immunohistochemistry labeling of vitamin D receptors. The atrium slices were deparaffinized with 100% xylol solution and graded ethanol (70%, 80%, 90%, 95%, and 100%), and rehydrated with distilled water and 1% bovine serum albumin (BSA, Sigma Aldrich) phosphate-buffered solution (blocking solution). The atrium slices were incubated with vitamin D receptor primary antibody (1:500, GTX104615, Genetex^®^) for 14–16 h at 4^o^C. After that, the slices were washed in blocking solution and incubated with the secondary antibody ImmunoHistoProbe Plus (Advanced Biosystems^®^) at room temperature for 30 min, according to the protocol suggested by the manufacturer. The slices were then stained with 3–3′-diaminobenzidine (DAB, Dako^®^) and counter-stained with Harris hematoxylin. Negative control slices were submitted to the same procedures, except the incubation with the vitamin D receptor primary antibody.

### 2.8 Euthanasia

At the end of the experiments, the animals were deeply anesthetized with an overdose of i.v. sodium thiopental (100 mg/kg, Cristalia, Itapira, SP, Brazil).

### 2.9 Statistical analysis

A Komolgorov-Smirnov test for normality was used for verifying the data distribution. Once the results fit to a normal distribution, they were expressed as mean ± S.E.M. MAP, HR, HRV, index of baroreceptor reflex, plasma calcium and calcitriol were compared among groups *via* one-way ANOVA, followed by Tukey posttest. Statistical analysis was conducted using the statistical software SPSS version 26. Significance level was set as *p* < 0.05.

## 3 Results

### 3.1 Heart rate variability analysis at resting in control rats or supplemented with different doses of vitamin D

#### 3.1.1 Linear analysis at frequency domain

As shown in Table 1, LF (abs) or (nu), and HF (abs) or (nu) of the VitD 15, 250, 3,750 UI/day groups (*n* = 6/group), respectively, showed similar values compared to control group (*n* = 6). In addition, LF/HF (abs)/(nu) ratio in the VitD 15, 250 and 3,750 IU/day were not different compared to the control group.

**TABLE 1:** Index of heart rate variability (HRV).

HRV	VitD-control	VitD 15 IU/day	VitD-250 IU/day	VitD-3,750 IU/day
Index-Frequency Domain				
LF (abs) (ms^2^)	6.43 ± 0.57	7.70 ± 0.38	9.73 ± 0.29	13.97 ± 0.14
HF (abs) (ms^2^)	22.79 ± 0.57	18.67 ± 0.38	28.25 ± 0.29	35.04 ± 0.14
Total Power (ms^2^)	29.22 ± 12.21	26.37 ± 8.59	38.01 ± 6.74	49.01 ± 3.48
LF (nu)	22.00 ± 4.64	29.20 ± 4.44	25.67 ± 4.27	28.50 ± 4.16
HF (nu)	78.00 ± 4.64	70.80 ± 4.44	74.33 ± 4.27	71.50 ± 4.16
LF/HF (abs)	0.28 ± 1.00	0.41 ± 1.00	0.35 ± 1.00	0.40 ± 1.00
LF/HF (nu)	0.31 ± 0.08	0.47 ± 0.10	0.40 ± 0.08	0.45 ± 0.10
LF/HF (abs)/(nu)	0.90 ± 12.50	0.87 ± 10.00	0.88 ± 12.50	0.89 ± 10.00
Index-Time Domain				
SDNN (ms)	3.35 ± 0.54	3.35 ± 0.42	3.54 ± 0.82	3.62 ± 0.47
RMSSDN (ms)	2.47 ± 0.85	1.87 ± 0.36	2.02 ± 0.39	2.27 ± 0.48
Non Linear Analysis				
Sample Entropy (nu)	1.72 ± 0.48	1.46 ± 0.48	1.43 ± 0.25	1.50 ±0.19
SD1 (nu)	1.75 ± 0.62	1.32 ± 0.26	1.43 ± 0.27	1.62 ± 0.33
SD2 (nu)	4.35 ± 0.81	4.52 ± 0.62	4.78 ± 1.27	4.85 ± 0.60
DFA - α1 (nu)	1.18 ± 0.29	1.45 ± 0.09	1.41 ± 0.08	1.36 ± 0.11
DFA - α2(nu)	0.63 ± 0.25	0.68 ± 0.18	0.75 ± 0.11	0.70 ± 0.16

Data are as mean ± SEM, *n* = 6/group. One-way ANOVA, followed by Tukey posttest. Significance level: *p* < 0.05. Abbreviations: abs, absolute (non-normalized); nu, normalized.

#### 3.1.2 Linear analysis at time domain

SDNN and RMSSD of the VitD 15, 250, and 3,750 UI/day groups (*n* = 6/group), respectively, showed similar values compared to control group (Table 1).

#### 3.1.3 Nonlinear analysis

No difference was observed in the sample entropy, SD1, SD2, short-term detrended fluctuations analysis (DFA-α1), and long-term detrended fluctuations (DFA-α2) values of the VitD 15, 250, and 3,750 UI/day groups (*n* = 6/group), respectively in comparison to the control group (*n* = 6) (Table 1).

### 3.2 Baroreceptor and cardiopulmonary (Bezold-Jarisch) reflex responses in control rats or submitted to supplementation with different doses of vitamin D

**FIGURE 1:**
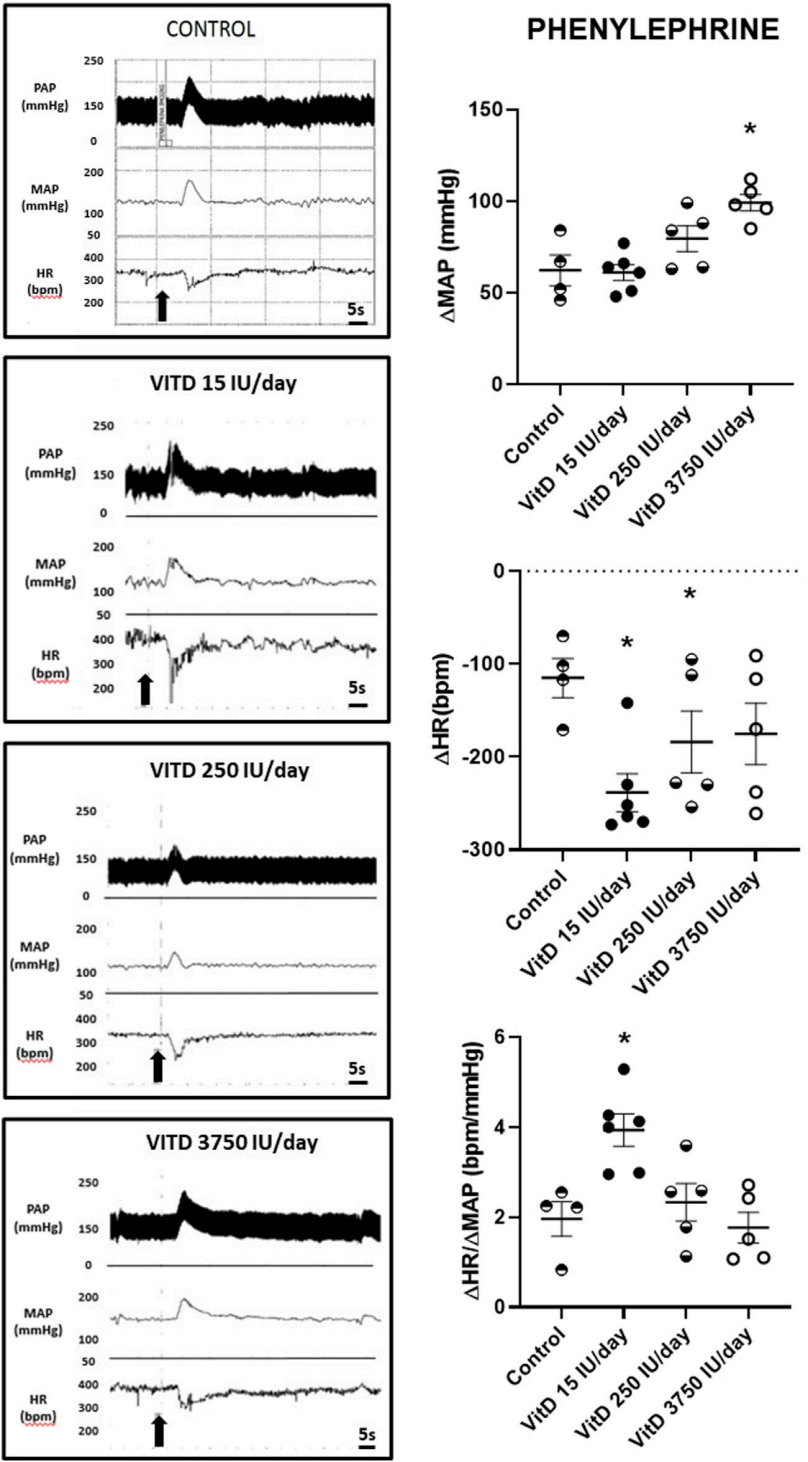
Left panels—Pulsatile arterial pressure (PAP), mean arterial pressure (MAP, mmHg), and heart rate (HR, bpm) tracings in Control, and Vitamin D 15, 250 and 3,750 IU/day treated-rats for 3 days in which phenylephrine (3 μg/kg of b.w.) was administrated in bolus injection intravenously. Right panels–Change in mean arterial pressure (ΔMAP, mmHg), heart rate (ΔHR, bpm), and index of baroreceptor reflex (ΔHR/ΔMAP in module, bpm/mmHg) evoked by a pressor dose of phenylephrine (3 μg/kg of b.w.) in Control rats and submitted to supplementation with different doses of Vitamin D (VitD 15, 250, 3,750 IU/day) for 3 days. (*n* = 4–6/group). One-way ANOVA followed by Tukey posttest. *p < 0.05 vs. Control.

Baseline (resting) MAP (123 ± 4, 123 ± 7, 123 ± 6 mmHg) and HR (373 ± 18, 334 ± 10, 334 ± 9 bpm) in the VitD 15, 250, and 3,750 UI/day groups, respectively, (*n* = 4–6/group) showed no difference compared to the Control group (118 ± 3 mmHg and 334 ± 13 bpm). Despite the intravenous injection of PHE (3 μg/kg of b.w.) elicited a similar pressor response in the rats VitD 15 (+61 ± 4 mmHg) and VitD 250 IU/day groups (+80 ± 7 mmHg) compared to Control group (+62 ± 8 mmHg), the reflex bradycardia was significantly greater in the VitD 15 (−239 ± 20 bpm) and Vit 250 IU/day groups (−184 ± 33 bpm) compared to the Control group (−115 ± 21 bpm). In contrast, the pressor response to PHE in VitD 3,750 IU/day group was significantly enhanced (99 ± 5 mmHg) compared to the Control group, whereas the reflex bradycardia (−175 ± 33 bpm) was not different compared to the Control group (Figure 1). Nevertheless, the index of the baroreceptor reflex tested with phenylephrine (3 μg/kg of b.w.) showed that rats supplemented with VitD 15 IU/day for 3 days had a significant increase in the sensitivity of the bradycardic component of the baroreflex (3.94 ± 0.36 bpm/mmHg) compared to Control rats (1.96 ± 0.38 bpm/mmHg) (*p* < 0.011). No difference was observed in the index of baroreceptor reflex tested with phenylephrine in rats treated with VitD 250 (2.33 ± 0.42 bpm/mmHg) and 3,750 IU/day (1.77 ± 0.34 bpm/mmHg) in comparison to the Control group.

**FIGURE 2:**
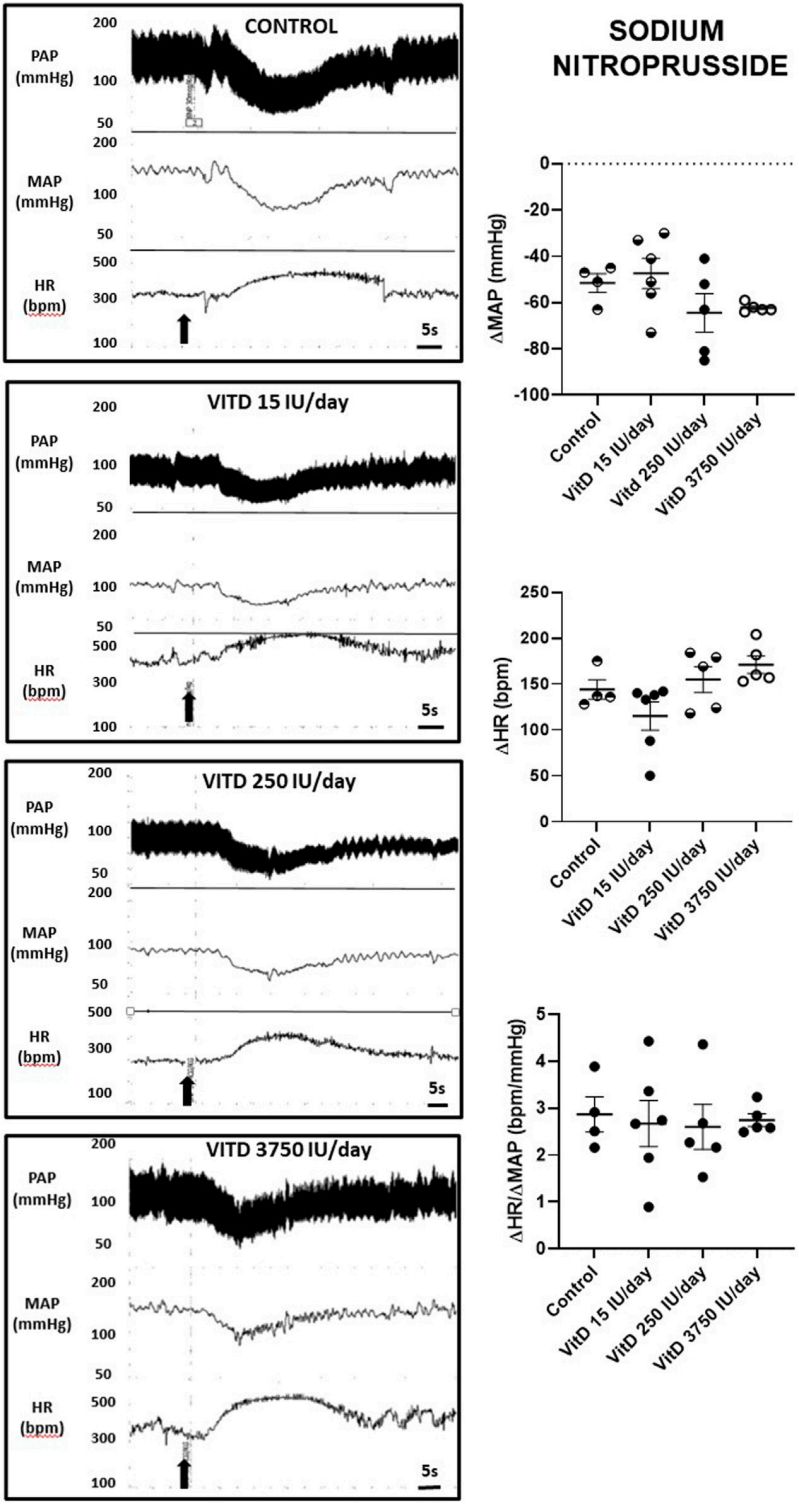
Left panels—Pulsatile arterial pressure (PAP), mean arterial pressure (MAP, mmHg), and heart rate (HR, bpm) tracings in Control, and Vitamin D 15, 250 and 3,750 IU/day treated-rats for 3 days in which sodium nitroprusside (30 μg/kg of b.w.) was intravenously injected in bolus. Right panels–Change in mean arterial pressure (ΔMAP, mmHg), heart rate (ΔHR, bpm), and index of baroreceptor reflex (ΔHR/ΔMAP in module, bpm/mmHg) yielded by a depressor dose of sodium nitroprusside (30 μg/kg of b.w.) in Control rats and submitted to supplementation with different doses of Vitamin D (VitD 15, 250, 3,750 IU/day) for 3 days. (*n* = 4–6/group). One-way ANOVA followed by Tukey posttest. *p < 0.05 vs. Control.

Similar hypotension and reflex tachycardia evoked by SNP was observed comparing the VitD 15 (−47 ± 6 mmHg and +115 ± 15 bpm), 250 (−64 ± 8 mmHg and +154 ± 14 bpm) and 3,750 IU/day treated rats (−62 ± 1 mmHg and +154 ± 14 bpm) compared to control group (−51 ± 4 mmHg and +171 ± 9 bpm) (*n* = 4–6/group) (Figure 2). The index of the baroreceptor reflex tested with sodium nitroprusside showed no difference in the sensitivity of tachycardic component of the baroreflex in rats supplemented with VitD 15 (2.68 ± 0.49 bpm/mmHg), 250 (2.60 ± 0.48 bpm/mmHg) and 3,750 IU/day (2.75 ± 0.14 bpm/mmHg) compared to the Control group (2.87 ± 0.37 bpm/mmHg).

**FIGURE 3:**
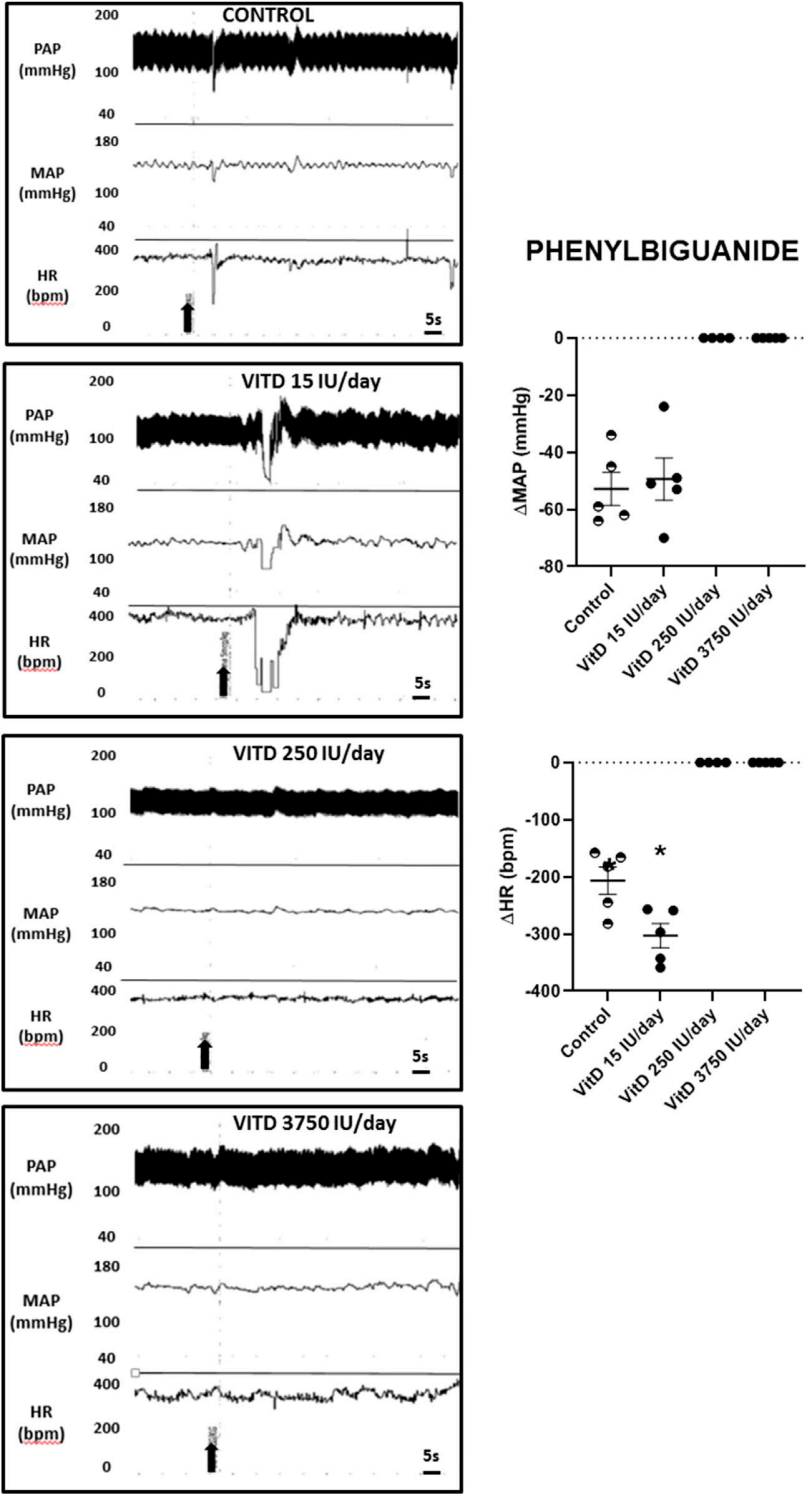
Left panels—Pulsatile arterial pressure (PAP), mean arterial pressure (MAP, mmHg), and heart rate (HR, bpm) tracings in Control, and Vitamin D 15, 250 and 3,750 IU/day treated-rats for 3 days in which phenybiguanide (5 μg/kg of b.w.) was administrated intravenously. Right panels–Change in mean arterial pressure (ΔMAP, mmHg) and heart rate (ΔHR, bpm) elicited by phenylbiguanide (5 μg/kg of b.w.) in Control rats and submitted to supplementation with different doses of Vitamin D (VitD 15, 250, 3,750 IU/day) for 3 days. (*n* = 4–6/group). One-way ANOVA.

Intravenous injection of PBG elicited similar hypotension in VitD 15 IU/day (−49 ± 7 mmHg) and Control groups (−53 ± 6 mmHg), nevertheless the bradycardia yielded by PBG was significantly enhanced in the VitD 15 IU/day group (−303 ± 21 bpm) compared to the control group (−207 ± 24 bpm). In the VitD 250 and VitD 3,750 IU/day groups, the PBG responses were abolished (Figure 3).

### 3.3 Plasma calcium and calcitriol dosage in control rats or supplemented with different doses of vitamin D

**FIGURE 4:**
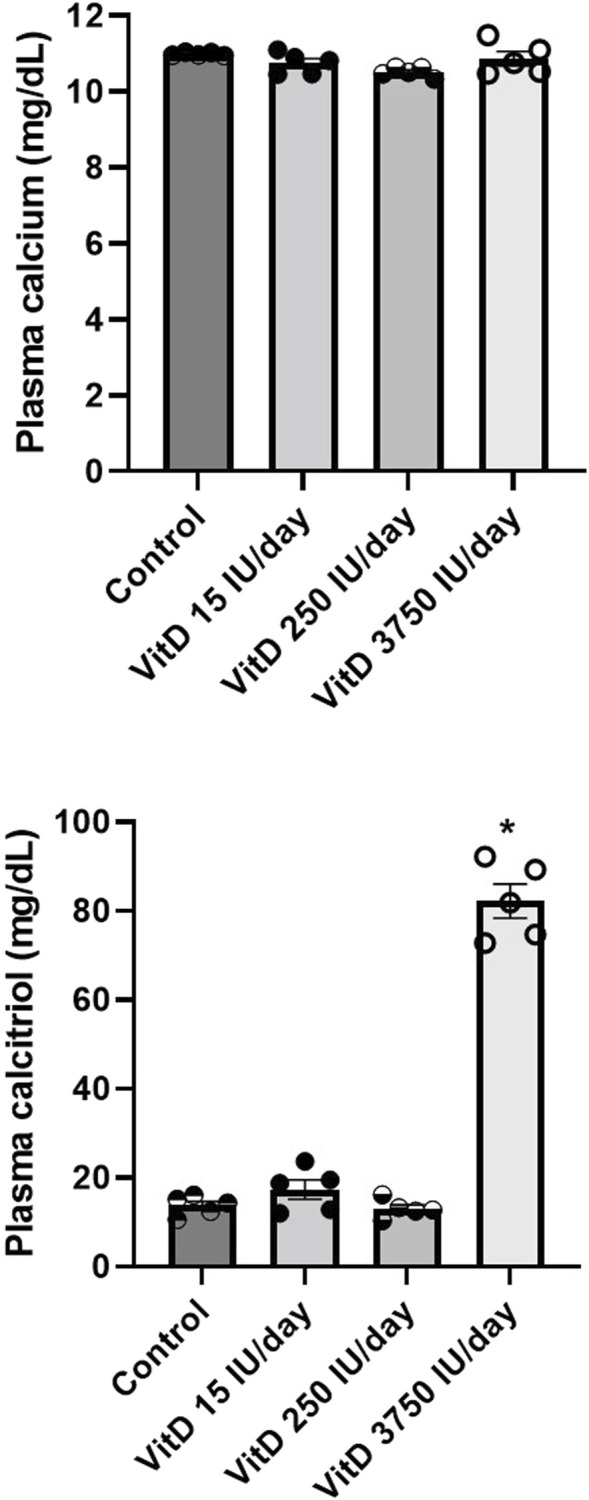
Plasma calcium (mg/dl) and calcitriol (µg/ml) in Control rats and submitted to supplementation with different doses of Vitamin D (VitD 15, 250, 3,750 IU/day) for 3 days. (*n* = 5–6/group). One-way ANOVA followed by Tukey posttest. *p < 0.05 vs. Control.

Plasma calcitriol dosage (*n* = 6/group) showed similar values comparing VitD 15 IU/day (17.38 ± 2.18 μg/ml), VitD 250 IU/day (13.11 ± 0.07 μg/ml) and control groups (13.76 ± 0.98 μg/ml), however, it was significantly higher in the VitD 3,750 IU/day (80.11 ± 3.75 μg/ml) compared to the control group (13.76 ± 0.98 μg/ml) (Figure 4).

Calcium serum levels were not different among VitD 15 IU/day (10.74 ± 0.12 μg/ml), 250 IU/day (10.41 ± 0.09 μg/ml) and 3,750 IU/day (10.97 ± 0.18 μg/ml) groups compared to control group (10.98 ± 0.01 μg/ml) (Figure 4).

### 3.4 Vitamin D receptor immunolabeling in the right atria of control rats or supplemented with different doses of vitamin D

The majority of the vitamin D receptors-immunolabeled were found in the cytoplasm of the right atria cells in rats supplemented with VitD 15, 250 and 3,750 IU, but also in the vehicle-treated rats. Only few cells were immunostained for vitamin D receptors in the nucleus of all groups of rats. Negative controls did not show any labeling for vitamin D receptors.

**FIGURE 5:**
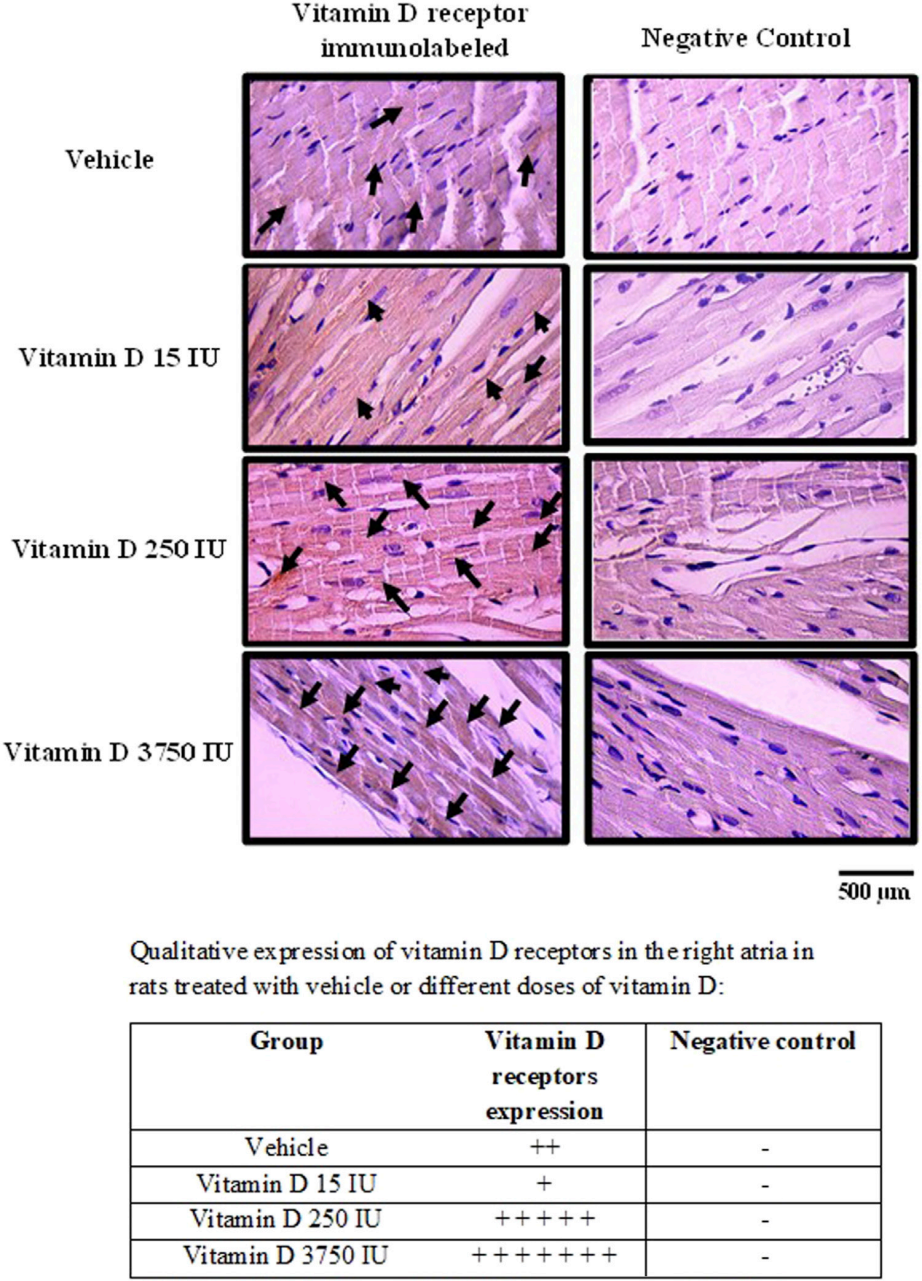
Photomicrograph of right atria slices showing vitamin D receptors-immunolabeled in the cells (left panel) in control rats (treated with vehicle) or supplemented with different doses do vitamin D (15, 250 or 3,750 IU) for 3 days. Arrows show the immunostaining of vitamin D receptors. Negative controls (right panels) show absence of labeling for vitamin D receptors. Amplification: 400X.

Despite the distribution of vitamin D receptors immunolabeled was irregular throughout the right atria and none pattern was not found in the slices, rats supplemented with VitD 15 IU showed less areas immunostained for vitamin D receptors in the right atria in comparison to the other groups. In contrast, a large area with almost all cells in the right atria were strongly immunostained for vitamin D receptors in rats supplemented with VitD 3,750 IU compared to the other groups (Figure 5).

## 4 Discussion

Our data showed that rats submitted to the supplementation with different doses of vitamin D (15, 250 and 3,750 IU/day) for 3 days show similar baseline MAP and HR compared to Control rats. Heart rate variability analysis in the frequency domain demonstrated that LF (nu), HF (nu) and LF/HF ratio in the vitamin D supplement groups are also similar to the Control group. The heart rate variability was also analyzed in time domain and by nonlinear analysis, which showed no difference between VitD supplemented and Control groups.

Although the baseline MAP and HR values show similarity among groups, the reflex bradycardia evoked by a pressor dose of PHE in bolus injection was enhanced in rats with VitD 15 and 250 IU/day supplementation for 3 days compared to Control rats. The highest dose of VitD (3,750 IU/day supplemented for 3 days) evoked a higher pressor response upon i.v. PHE, nevertheless, the reflex bradycardia was not different from Control rats. Despite these changes in MAP and HR yielded by phenylephrine, the index of the baroreceptor reflex showed a greater sensitivity of the bradycardic component of the baroreflex in rats supplemented with VitD 15 IU/day for 3 days compared to Control rats. VitD 250 and 3,750 UI/day groups presented similar index of the baroreceptor reflex tested with phenylephrine. These findings suggest that the treatment with VitD 15 IU/day for 3 days could improve the sensitivity of the bradycardic component of the baroreflex, nevertheless, the higher doses (Vit 250 and 3,750 IU/day groups) supplemented for 3 days were unable to improve it, causing similar responsiveness in comparison to the Control group. It is still uncertain at which level of the baroreceptor reflex circuitry the VitD at the lowest dose that we used in the current study is producing any change in order to improve the bradycardic component of the baroreflex. Either the activity of the baroreceptors localized in the aorta and carotid arteries, or neurons in the medullary areas of the circuitry involved in the baroreceptor reflex control as the nucleus of the solitary tract, and nucleus ambiguus could be affected, consequently affecting the parasympathetic autonomic efferents (Colombari et al., 2001; Cravo et al., 2009). In addition, another hypothesis would be the heart atria cells that could be targeted by VitD in the lowest dose of this study, as these cells contains the muscarinic receptors for the acetylcholine released by the parasympathetic efferents. Further studies are still necessary to investigate if the expression of muscarinic receptors in the heart cells could be affected by Vit D supplementation, as well as if the gene expression of these receptors in the cardiac cells is altered in rats treated with VitD. In addition, the possible change in the neurotransmission in the vagal preganglionic neurons in the nucleus ambiguous of the medulla oblongata in rats supplemented with VitD still require more investigation.

In contrast, our data showed that the tachycardic component of the baroreflex elicited by a bolus intravenous injection of a depressor dose of SNP had no difference comparing rats which underwent supplementation with different doses of vitamin D and Control animals, which is suggestive of a selective action of the vitamin D interfering in the bradycardic component, but not in the sympathetic component of the baroreceptor reflex.

Our data showed an absence of changes in the heart rate variability under resting conditions in rats supplemented with different doses of VitD. In contrast, baroreceptor reflex responses elicited by PHE in rats treated with the lowest dose of VitD (15 IU/day for 3 days) and the Bezold-Jarisch reflex responses were affected by supplementation with VitD at different doses. These findings suggest that VitD supplementation in different doses (15, 250 and 3,750 IU/day for 3 days) in rats does not show changes in the cardiac autonomic modulation under resting conditions, nevertheless, upon stimulation of the cardiovascular system, the responsiveness is altered, particularly the sensitivity of the barorereflex (bradycardic component) in rats treated with VItD 15 IU/day for 3 days and the responses of the cardiopulmonary reflex (Bezold-Jarisch) in all doses of VitD supplemented in this study.

Interestingly, our findings also demonstrated that the cardiopulmonary (Bezold-Jarisch) reflex responses were paradoxal in rats supplemented with VitD 15 IU/days in comparison to rats supplemented with 250 and 3,750 IU/day for 3 days. Rats which received VitD 15 IU/day presented exaggerated bradycardic responses to PBG compared to control rats despite the similar hypotension in both groups. The bradycardic response to PBG is dependent on activation of vagal efferents to the heart, whereas the hypotensive response to PBG depends on sympathetic inhibition (Mark, 1983). Either in the Bezold-Jarisch reflex or in the baroreceptor reflex response evoked by PHE, the responses dependent on activation of the vagus efferents to the heart were significantly more intense in rats supplemented with VitD 15 IU/day for 3 days. Conversely, the supplementation with VitD 250 and 3,750 IU/day for 3 days completely abolished the Bezold- Jarisch reflex, suggesting that either the sympathetic inhibition or the vagal activation dependent responses were affected by the supplementation with these doses of VitD. Previous studies have suggested that vitamin D can exert genomic and non-genomic actions (Zmijewski and Carlberg, 2020). The last ones have not been attributed to vitamin D actions at nucleus of cells and maybe due to actions in potential membrane-bound or intracellular targets in order to provide rapid responses to the stimulus. In primary-cultured myocytes isolated from chicken embrionary heart, it has been demonstrated that vitamin D increases calcium influx mediated by cAMP, suggesting a G protein-coupled membrane receptor effect (De Boland and Boland, 1994). In addition, rapid non-genomic activity of vitamin D was also confirmed *in vivo* in chickens (Nemere et al., 2004). Phenylbiguanide binds to 5-HT3 receptors that are coupled to ion channels permeable to Na^+^ and Ca^++^, leading to rapid depolarization due to increase in cytosolic calcium (Rahman, 2011). Indeed, in the current study, a hypothesis of a possible interference of vitamin D on 5-HT3 receptor signaling in the heart atrial cells could explain the enhanced reflex response to phenylbiguanide in rats supplemented with vitamin D 15 IU/day for 3 days. Despite earlier studies have demonstrated that vitamin D treatment increases mRNA of tryptophan hydroxylase and monoaminoxidase in neurons (Jiang et al., 2014), it is unknown if the supplementation with vitamin D in eutrophic rats can also modulate the gene transcription of ion channels or protein kinase A, which can modulate ion channels in the heart atrial cells. In the present study, VitD at 250 and 3,750 IU/day supplemented for 3 days abolished the reflex responses evoked by phenylbiguanide. The hypothesis that vitamin D could modulate ion channels or protein transcription in atrial cells, which could impair depolarization, would explain the abolishment of Bezold-Jarisch reflex responses with phenylbiguanide injections. However, this hypothesis still require further investigation.

In spite of the abnormal responsiveness of the vagal component of the baroreflex or in the Bezold-Jarisch reflex, these alterations do not seem to be related to changes in plasma calcium levels. The supplementation with VitD 15, 250 and 3,750 IU/day for 3 days were unable to produce differences in the plasma calcium compared to control rats, and only in VitD 3,750 IU/day supplemented rats, the plasma calcitriol levels, which is the active form of Vit D, were higher compared to control rats.

In the present study, immunolabeling of vitamin D receptors in right atria demonstrated that the majority of these receptors were localized in the cytosol of the heart atrial cells with larger areas of cells labeled in rats supplemented with 3,750 IU/day for 3 days. Vitamin D receptors can be located in the cytoplasm or in the nucleus of cells (Zmijewski and Carlberg, 2020). In the classic vitamin D pathway, vitamin D penetrates the cell membrane as a free molecule or bound to DBP, and it is transported *via* endocytosis mediated by the LRP2-CUBN complex present in the cell membrane. Then, the VitD binds to the vitamin D- Retinoid X receptor complex present in the cytosol or in the nucleus resulting in modulation of the target gene expression (Zmijewski and Carlberg, 2020). Interestingly, in rats supplemented with 15 IU/day, we found less areas in the right atria immunolabeled for vitamin D receptors. Earlier studies have shown that vitamin D receptors can underwent proteasome-mediated degradation in a vitamin D dependent-manner in order to downregulate the vitamin D-activated transcriptional response (Masuyama and MacDonald, 1998). Such evidence strengthens our finding as the lower dose of vitamin D (15 IU) supplemented in the animals has already activated the receptors and likely accomplished the functional role of gene transcription. In contrast, we observed that rats supplemented with 250 and 3,750 IU/day showed larger areas of cells immunostained for vitamin D receptors. Previous reports have shown that CD4^+^ T-cells can produce 1,25(OH)2D3, the active form of vitamin D3, which stabilizes the vitamin D receptors by upregulation, due to increase in the protein expression of vitamin D receptors, protecting it from proteasomal degradation (Kongsbak et al., 2014). Indeed, we hypothesized that higher doses of vitamin D supplemented in the animals in the current study could be upregulating the vitamin D receptors in the right atria. Nevertheless, the transcriptional mechanisms activated by vitamin D supplemented with higher doses of vitamin D are likely affecting the synthesis of proteins in the right atria cells, which should have a pivotal role in the Bezold-Jarisch reflex responses.

In the current study, we have used the beat-by-beat pulse interval of arterial blood pressure for measurement of heart rate variability, which could be a limitation of this study as the electrocardiogram (ECG) recordings have been considered as the gold-standard method for acquiring data for the later generation of beat-to-beat time series. Nevertheless, the HRV analysis is also feasible by utilizing pulsatile arterial pressure recordings. Studies of Dias et al. (2016) in rats have tested these interactions and showed a strong correlation comparing cardiac intervals obtained from RR intervals vs. systolic intervals and an even stronger correlation between cardiac intervals obtained from RR intervals vs. diastolic intervals. The time and frequency domain analysis showed slight differences comparing results obtained from RR intervals vs. systolic intervals, whereas no differences were observed between data obtained from RR intervals vs. diastolic intervals (Dias et al., 2016).

We have recorded the arterial pressure, heart rate, and reflexes in freely moving animals in the present study 24-h recovered from surgeries, which could lead to inquire whether the animals were stressed or not post-surgery and considered a limitation of this study. However, we would like to emphasize that all the surgeries were performed by well trained researchers in our lab and the animals showed a very good recovery after the procedures. This can be also confirmed by baseline arterial pressure and heart rate, which were within the normal acceptable values and indicate that the animals were not stressed when we performed the recordings similarly to previously shown in other studies (Colombari et al., 1994; Colombari et al., 1996; Sato et al., 1999; Sato et al., 2000; Akemi Sato et al., 2001; Viera et al., 2007; Giusti et al., 2011).

In conclusion, our findings suggest that the supplementation with different doses of VitD (15, 250 and 3,750 IU/day) for 3 days did not affect the resting arterial pressure, heart rate and autonomic modulation on the heart in rats. In spite of that, the supplementation with the lowest dose of VitD (15 IU/day for 3 days) in this study improved the sensitivity of the bradycardic component of the baroreflex, whereas higher doses of supplementation with VitD (250 and 3,750 IU/day for 3 days) were unable to cause such effect. Further, the Bezold-Jarisch reflex responses were affected regardless the dose of VitD (15, 250 or 3,750 IU/day) supplementation for 3 days in rats, suggesting that responses dependent on stimulation of the cardiovascular system could be impacted by VitD supplementation.

## Data Availability

The original contributions presented in the study are included in the article/Supplementary Material, further inquiries can be directed to the corresponding author.
